# A Spontaneous Bilateral Quadriceps Tendon Rupture in a Patient Undergoing Long-Term Hemodialysis

**DOI:** 10.7759/cureus.36059

**Published:** 2023-03-13

**Authors:** Yassine Allata, Basmat Amal Chouhani, Ghita El Bardai, Nadia Kabbali, Tarik Sqalli Houssaini

**Affiliations:** 1 Nephrology, Dialysis, and Transplantation, Hassan II University Hospital, Fez, MAR; 2 Laboratory of Epidemiology and Research in Health Sciences, Faculty of Medicine, Pharmacy and Dentistry, Sidi Mohamed Ben Abdellah University of Fes, Fez, MAR

**Keywords:** tendon rupture, β2 microglobulin amyloidosis, quadriceps tendons, hyperparathyroidism, dialysis

## Abstract

Spontaneous quadriceps tendon rupture is very rare. Its occurrence is usually linked to an underlying disease that weakens the tendons causing them to rupture. Here, we report the case of a 44-year-old patient undergoing long-term hemodialysis who had spontaneous bilateral quadriceps tendon rupture. We present the clinical presentation and the management of this injury.

## Introduction

The extensor apparatus of the knee is a functional and anatomical entity made up of the femoral quadriceps muscle, the quadriceps tendon, the patella, and the patellar tendon linking it to the tibial tuberosity. The soft tissue structures provide both static and dynamic stability to the patella [1].

The rupture of the quadriceps tendon is defined by a solution of continuity of the chain of transmission of the extension of the leg on the thigh.

The mechanisms of quadriceps tendon tear can be a rapid and unusual muscle contraction with a planted foot and a partially flexed knee, direct localized trauma or tendon tear, or a fall with a flexed knee [2]. A complete rupture of this tendon results in loss of muscle function and the ability to stand or walk. Bilateral quadriceps tendon rupture is a relatively rare presentation with very few reported cases in the literature.

To date, the pathophysiological mechanisms of tendon ruptures are not entirely understood. One of the suggested reasons was the vascular compromise or the disruption of the tendon structure triggered by systemic conditions. Degenerative changes, mainly tendon lipomatosis, were the most common finding in the pathology assessment of spontaneously ruptured tendons making them its most probable cause [3,4]. Several other risk factors tend to weaken the tendon, predisposing it to rupture.

In chronic renal disease (CKD), especially in patients on long-term hemodialysis, the pathogenesis remains unclear but three factors are particularly involved, namely, degeneration caused by chronic acidosis leading to the deposition of elastin in the tendons; secondary hyperparathyroidism which weakens the bone-tendon junction (increased bone resorption); and β2 microglobulin amyloidosis which accumulates, in particular, in the joints, bones, and tendons resulting in decreased tendon elasticity [1,5].

## Case presentation

A 44-year-old patient, suffering from CKD of undetermined origin and undergoing long-term hemodialysis since 2013 (two five-hour sessions per week using a 1.8 m^2^ low-flux dialyzer), with a history of secondary hyperparathyroidism and vascular and soft tissue calcifications, presented on the day of his hemodialysis session complaining of weakness in both lower limbs. Prior to this incident, the patient was ambulatory and could walk with the aid of a cane. The patient did not report any fall or trauma. According to the patient, after waking up in the morning, he was unable to stand up and felt intense pain in the knees every time he tried to.

Clinical examination revealed an absolute deficit of active extension involving both knees, with the impossibility of support on both lower limbs and a suprapatellar hiatus with subcutaneous protrusion of the femoral trochleae. There was no sensitive deficit. The tendon reflexes were not assessed because of the painful palpation of the knees.

**Figure 1:**
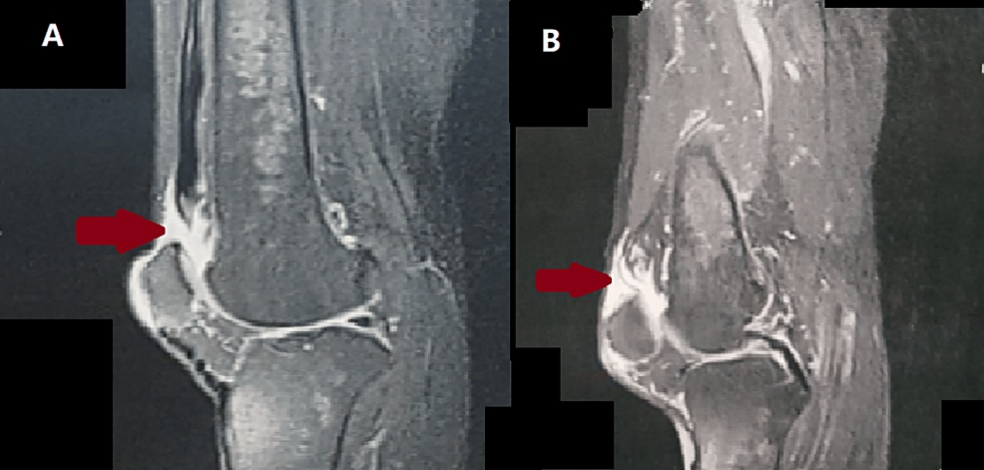
MRI sagittal section of the right knee (A) and left knee (B) showing a rupture of both quadriceps tendons.

The same-day MRI found a bilateral quadriceps tendon rupture with tendon retraction of approximately 20 mm. In addition, we noted an intra-articular effusion and edema of the soft tissue surrounding the quadriceps tendon (Figure 1).

His laboratory findings are presented in Table 1.

**Table 1:** Patient’s laboratory results.

	Results (reference range)
Serum creatinine	110 mg/L (6–12 mg/L)
Blood urea nitrogen	0.90 g/L (0.07–0.3 g/L)
Calcium	85 mg/L (85–105 mg/L)
Albuminemia	34 g/L (31–43 g/L)
Phosphatemia	93 mg/L (25–45 mg/L)
Bicarbonates	18 mmol/L (20–32 mmol/L)
C-reactive protein	15 mg/L (<9 mg/L)
Parathyroid hormone	1,953 pg/mL (<68 pg/mL)
Vitamin D	11.7 ng/mL (20–40 ng/mL)
β2 microglobulin	59 mg/L (1.1–2.4 mg/L)

The surgical management consisted of an end-to-end suture using X-shaped stitches after the evacuation of a local hematoma. The two operated limbs were immobilized with a splint keeping the knees in extension for six weeks. Passive rehabilitation was started early, but active rehabilitation was not allowed until after the sixth week and was continued for three months. The patient resumed normal activity at the end of rehabilitation.

Medical management focused on the treatment of secondary hyperparathyroidism. First, treating the hyperphosphatemia by combining a low phosphorus diet with a non-calcium phosphorus binder (sevelamer 2,400 mg/day). Subsequently, the optimization of dialysis by increasing the dialysis dose from 10 hours/week to 15 hours/week divided into three sessions (an initial regime of 18 hours/week had to be reduced after three months because of low patient compliance) and using a low calcium dialysate (calcium concentration of 1.25 mmol/L). The vitamin D deficiency was corrected using cholecalciferol (26 ng/mL), and after controlling hyperphosphatemia (56 mg/L), an active form (alfacalcidol) was used in the hope of controlling hyperparathyroidism.

Eleven months later, a parathyroidectomy was scheduled after an insufficient decline of the parathyroid hormone (PTH) (1,760 pg/mL) using available medical means (calcimimetics were not used due to financial reasons).

## Discussion

The fast diagnosis of bilateral quadriceps tendon ruptures is vital because early surgical intervention is an important prognostic factor, allowing both end-to-end tendon repair and optimization of tendon-bone anchorage [6]. Although difficult, this diagnosis should always be considered whenever there is an unexplained and sudden motor deficit of the lower limbs. The presence of hemarthrosis and pain can make it even harder to identify typical suprapatellar defects [2]. In such cases, imaging techniques such as ultrasound and MRI can confirm the diagnosis [7,8].

This case is the prime example of why one should not neglect the treatment of the underlying causes, such as secondary hyperparathyroidism and inadequate dialysis. The 2017 Kidney Disease Improving Global Outcomes guidelines on CKD-mineral and bone disorders recommended a treatment strategy based on the assessment of phosphate, calcium, and PTH levels [9].

The phosphate level should be kept close to the upper level because a low level can be predictive of malnutrition. Adequate dietary control of phosphate intake can help achieve this goal. Favoring proteins of vegetal origin over animal ones and avoiding inorganic phosphate present in processed food can help lower phosphate intake without impacting the nutritional status of the patient. Additionally, prescribing phosphate-lowering medication is often necessary because of the high phosphate intake and its low clearance when using conventional dialysis methods [10]. Lastly, optimizing dialysis parameters can help increase phosphate clearance by increasing the length and the number of sessions and favoring high-flux dialyzers when possible [11].

The calcium level should be kept at a normal range using calcium and vitamin D supplements when needed, and special care should be taken to avoid hypercalcemia and the associated vascular and soft tissue calcification [12].

The PTH level should be monitored and interpreted at the same time as the calcium and phosphate due to the tight link between them. In fact, the control of calcium and phosphate levels alone can lower the PTH to acceptable levels (two to nine folds the upper normal limit [13]). The use of calcimimetic (cinacalcet), calcitriol, and vitamin D analogs can help further lower the PTH. Parathyroidectomy still conserves its indication when the aforementioned drugs fail [14].

The β2 microglobulin accumulation in dialysis patients was not only linked to dialysis-related amyloidosis but was also responsible for increased mortality in this population [15]. While more efficient β2 microglobulin removal using high-flux membranes and hemofiltration was possible, it failed to demonstrate an improved outcome in randomized controlled trials [15,16]. Because the residual renal function is the only major determinant of β2 microglobulin level in the dialysis population, its preservation should be prioritized [17].

## Conclusions

The spontaneous rupture of both quadriceps tendons is very rare and usually the result of a systemic disease. The quick diagnosis and surgical treatment of this tendon tear are vital to achieving a full recovery. In our case, the clinical presentation suspected the diagnosis and the MRI confirmed the tendon rupture. The same-day surgical repair of the tear and the early rehabilitation helped our patient recover the full motor function of both limbs. The management of the underlying disease, namely, hyperparathyroidism and contributing factors of metabolic acidosis and inadequate dialysis, was needed to prevent further complications.

## References

[1] Grecomoro G, Camarda L, Martorana U (2008). Simultaneous chronic rupture of quadriceps tendon and contra-lateral patellar tendon in a patient affected by tertiary hyperparatiroidism. J Orthop Traumatol.

[2] Lim CH, Landon KJ, Chan GM (2016). Bilateral quadriceps femoris tendon rupture in a patient with chronic renal insufficiency: a case report. J Emerg Med.

[3] Kannus P, Józsa L (1991). Histopathological changes preceding spontaneous rupture of a tendon. A controlled study of 891 patients. J Bone Joint Surg Am.

[4] Ilan DI, Tejwani N, Keschner M, Leibman M (2003). Quadriceps tendon rupture. J Am Acad Orthop Surg.

[5] Pei YC, Hsieh PC, Huang LZ, Chiang CK (2011). Simultaneous bilateral quadriceps tendon rupture in a uremic patient. Formosan J Musculoskeletal Disord.

[6] Ciriello V, Gudipati S, Tosounidis T, Soucacos PN, Giannoudis PV (2012). Clinical outcomes after repair of quadriceps tendon rupture: a systematic review. Injury.

[7] LaRocco BG, Zlupko G, Sierzenski P (2008). Ultrasound diagnosis of quadriceps tendon rupture. J Emerg Med.

[8] Bhole R, Johnson JC (1985). Bilateral simultaneous spontaneous rupture of quadriceps tendons in a diabetic patient. South Med J.

[9] Ketteler M, Block GA, Evenepoel P (2017). Executive summary of the 2017 KDIGO Chronic Kidney Disease-Mineral and Bone Disorder (CKD-MBD) Guideline Update: what's changed and why it matters. Kidney Int.

[10] Cannata-Andía JB, Fernández-Martín JL, Locatelli F (2013). Use of phosphate-binding agents is associated with a lower risk of mortality. Kidney Int.

[11] Gross P, Six I, Kamel S, Massy ZA (2014). Vascular toxicity of phosphate in chronic kidney disease: beyond vascular calcification. Circ J.

[12] Tentori F, Blayney MJ, Albert JM (2008). Mortality risk for dialysis patients with different levels of serum calcium, phosphorus, and PTH: the Dialysis Outcomes and Practice Patterns Study (DOPPS). Am J Kidney Dis.

[13] Cozzolino M (2018). CKD-MBD KDIGO guidelines: how difficult is reaching the 'target'?. Clin Kidney J.

[14] Trombetti A, Stoermann C, Robert JH, Herrmann FR, Pennisi P, Martin PY, Rizzoli R (2007). Survival after parathyroidectomy in patients with end-stage renal disease and severe hyperparathyroidism. World J Surg.

[15] Okuno S, Ishimura E, Kohno K (2009). Serum beta2-microglobulin level is a significant predictor of mortality in maintenance haemodialysis patients. Nephrol Dial Transplant.

[16] Grooteman MP, van den Dorpel MA, Bots ML (2012). Effect of online hemodiafiltration on all-cause mortality and cardiovascular outcomes. J Am Soc Nephrol.

[17] Roumelioti ME, Nolin T, Unruh ML, Argyropoulos C (2016). Revisiting the middle molecule hypothesis of uremic toxicity: a systematic review of beta 2 microglobulin population kinetics and large scale modeling of hemodialysis trials in silico. PLoS One.

